# Recent Potential Noninvasive Biomarkers in Necrotizing Enterocolitis

**DOI:** 10.1155/2019/8413698

**Published:** 2019-04-22

**Authors:** Kewei Wang, Guozhong Tao, Zhen Sun, Karl G. Sylvester

**Affiliations:** ^1^Department of Gastrointestinal Surgery, The First Hospital of China Medical University, Shenyang, 110001 Liaoning Province, China; ^2^Department of Surgery, Stanford University School of Medicine, Stanford, CA 94305, USA

## Abstract

Necrotizing enterocolitis (NEC) is a rare but devastating gastrointestinal disease that predominately affects preterm neonates. Numerous studies have revealed that NEC is strongly associated with very low birth weight, degree of prematurity, formula feeding, infection, hypoxic/ischemic injury, and enteric dysbiosis. Given these clinical associations, the search for a deeper understanding of disease pathogenesis has led to an intense interest in the discovery and development of noninvasive biomarkers of NEC from stool, urine, and serum. Biomarkers for NEC may serve at least two general purposes of urgent unmet need: to improve diagnostic accuracy and disease prediction and to reveal the mechanism of the disease. This review will provide an overview of recent research focused on clinical NEC and highlight the advances that were made within the past five years towards the development of noninvasive diagnostic biomarkers.

## 1. Introduction

All premature infants are at risk of necrotizing enterocolitis (NEC), but incidence and mortality rates differ significantly throughout the world. In the United States alone, the incidence rate for NEC varies between 3 and 10% annually across most series with a mortality of up to 33% [1] and at an estimated cost to the US healthcare system of more than 1 billion dollars [2]. In the United Kingdom (UK), 163 English neonatal departments prospectively collected information on 118,073 newborns over two years and reported on 531 infants (0.4%) who developed severe NEC with a mortality of 48% [3]. In China, the incidence rate of NEC is 4.5% and 2.5% in very low-birth weight (VLBW, birth weight < 1500 g) and low-birth weight (LBW, birth weight < 2500 g) neonates, respectively, and the mortality rate of NEC at stages II and III was 41.7% [4].

The pathogenesis of NEC is incompletely understood and appears to be multifactorial. Current leading models of NEC pathogenesis purport that NEC may initiate due to a maladaptive immune responses to a dysbiotic ecosystem in the preterm gut [5, 6]. Potential risk factors for NEC include very low birth weight [7], prematurity [8], formula feeding [9, 10], hypoxic/ischemic insults [11], infection [12], and microbial dysbiosis [13–15]. Several recent reviews have primarily focused on the pathogenesis of NEC [16, 17]. The clinical diagnosis of NEC is currently made based on a combination of clinical, laboratory, and radiologic findings. The challenge remains the patient's clinical symptoms, and imaging findings may appear late such that a potential therapeutic window to prevent disease progression may be quite narrow. Therefore, there is an urgent need for identification of noninvasive biomarkers that are suitable for early diagnosis of NEC that may provide the opportunity for earlier intervention and disease progression mitigation. In the past 5 years, there has been a significant increase in research efforts focusing on the discovery of noninvasive diagnostic biomarkers. To summarize this experience, we searched the literature in the MEDLINE and PubMed databases from January 2014 to September 2018 using the following key words: biomarker, diagnosis, and necrotizing enterocolitis. For this review, we excluded the literature about etiology and treatment of NEC as well as the literature about markers in experimental NEC that did not include an examination of human tissue or samples. This review will highlight these advances in clinical biomarkers of NEC.

## 2. Biomarkers

**Figure 1:**
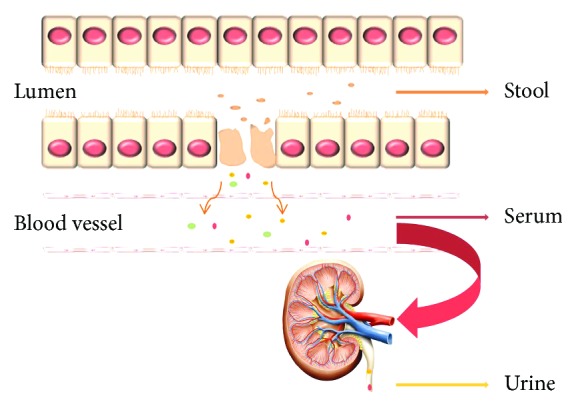
Source of the noninvasive biomarker for NEC. When intestinal epithelial cells are damaged, some cell component can be detached, mixed with the feces, and then excreted. Some proteins or cytokines are released into the bloodstream and then excreted by the kidneys.

The clinical application of biomarkers may include surveillance, early diagnosis, predicting severity and prognosis of disease, or response to therapy. The consensus among experts is that biomarkers may find the greatest immediate utility in providing for an early diagnosis of NEC or for identifying those premature infants most at risk of NEC prior to overt clinical manifestations. The rationale for this framework is that early or preclinical disease recognition will provide the greatest possible opportunity for disease prevention or mitigation. Since the intestine and colon cannot be directly sampled, research has focused on the development of noninvasive measures for NEC biomarkers [18]. From a practical perspective, there are multiple approaches to noninvasive interrogation including the sampling of neonatal stool, urine, and serum (Figure 1). The most expansive experience has been acquired among studies that have utilized fecal, urine, or serum biomarkers that can contribute to the diagnosis of NEC [19]. Herein, we provide for a review of the more promising noninvasive biomarker descriptions from the past five years and include a description of their biologic relevance and possible clinical utility that is summarized in Table 1.

### 2.1. Biomarkers in Stool

#### 2.1.1. Calprotectin

Increased fecal calprotectin indicates the increased presence of neutrophils in the intestinal epithelium, which can occur during intestinal inflammation. Prior studies have found that an increase in calprotectin in the preterm neonate is associated with an early diagnosis of NEC and prediction of disease severity [20–22]. Bin-Nun et al. measured fecal calprotectin in neonates with a clinical suspicion of NEC using a rapid calprotectin assay. The authors calculated a cut-off value (480 *μ*g/g) of fecal calprotectin that gave a maximum sum of diagnostic sensitivity and specificity (sensitivity 100% and specificity 84.6%) [23]. Pergialiotis et al. conducted a systematic review of fecal calprotectin levels as a noninvasive marker for NEC. This review included 13 studies of 601 neonates and showed that the sensitivity and specificity of fecal calprotectin as a diagnostic marker were 76-100% and 39-96.4%, respectively [24]. However, other scholars have reported no difference in fecal calprotectin levels between NEC and control groups and further concluded that fecal calprotectin would not be useful for early NEC diagnosis given large individual variation in fecal levels [25]. Therefore, more data is needed to determine whether a sudden increase in fecal calprotectin can be used as a diagnostic or prognostic biomarker before clinical symptoms occur. Results from recent research showed that 62% of preterm neonates have a high baseline calprotectin (≥200 *μ*g/g) level throughout the first week after birth [26]. There were also a significant positive linear relationship in the gestationalage < 26weeks group and a significant negative linear relationship in the gestationalage ≥ 26weeks and <30 weeks group between the fecal calprotectin concentration and days after birth [27]. Stool from preterm neonates has a higher range of calprotectin than stool from term neonates. Thus, a calprotectin upper reference interval that incorporates corrected gestational age may help in predicting the onset of NEC more accurately [28]. In summary, fecal calprotectin may be an effect biomarker to screen for NEC among subsets of at-risk neonates. However, since calprotectin is an inflammatory marker and therefore not specific for NEC, an approach combining it with other composite measures may increase its specificity. Additional prospective well-controlled trials are needed to verify utility and increase specificity.

#### 2.1.2. Microbiota

In recent years, microbiota has been found to be crucial for metabolic, hormonal, and immunologic homeostasis of their hosts. Microbial composition of stool is different between NEC and non-NEC cases, with a greater abundance of Proteobacteria among NEC infants [29]. The samples used for analysis were obtained before the diagnosis of NEC. These results were not therefore affected by the therapeutic effect (antibiotics) and can more accurately reflect microbial composition preceding a diagnosis. Still, the precise contribution to NEC pathophysiology remains ambiguous. There was an increase in total bacterial count (9.8-fold) in affected newborns 24 hours before the occurrence of clinical manifestations of NEC primarily due to the expansion of E. coli species (21.6-fold). However, intriguingly, the microbiota composition did not significantly differ from that of extremely low-birth weight (ELBW) neonates 5 days before the occurrence of NEC [30]. These results suggest that if the total number of bacteria increases significantly, the newborn may suffer from NEC within a short period. Currently, it is unknown if this type of assessment can have practical clinical applications given several technical and biologic limitations.

#### 2.1.3. Fecal Volatile Organic Compounds (VOCs)

Fecal VOCs may reflect not only gut microbiota composition but also their metabolic activity and interaction with the host. Identification of “disease-specific” VOCs may increase the understanding of pathophysiological mechanisms and may assist in the development of a useful biomarker [31]. A recent study showed that fecal VOCs of infants with NEC are significantly different from those of nonill infants and infants with sepsis 2 or 3 days prior to the occurrence of clinical manifestations of NEC [32]. These findings, while offering a potentially compelling new line of investigation, included only 13 samples from each group, thus indicating the need and necessity to expand the sample size further to verify its efficacy as a biomarker for NEC.

### 2.2. Biomarkers in Urine

Due to its rich source of peptides and proteins, urine may contain many potential biomarkers that can be easily and noninvasively collected [33].

#### 2.2.1. Intestinal Fatty Acid-Binding Protein (I-FABP)

I-FABP is one of the most widely studied potential biomarkers of NEC. I-FABP is released into the bloodstream upon intestinal injury and is excreted by the kidneys. Thus, I-FABP can be detected in either blood or urine as a potential biomarker of intestinal mucosal damage caused by NEC [34]. Gollin et al. reported that elevated I-FABPu (in urine) was a sensitive and specific predictive biomarker for NEC one day before clinical manifestations [35]. Levels of urinary I-FABP is higher in NEC patients than in sepsis patients or healthy infants with a sensitivity of 81% and a specificity of 100% [36]. It has also been observed that the length of intestinal resection in surgical NEC was closely related to serum or urinary I-FABP levels at the occurrence of the disease [37]. Moreover, I-FABPu correlated significantly with serum IL-6 and lactate during the first eight hours of the disease [38]. Together, these results suggest that I-FABP may be a clinically valid biomarker for NEC in the future. But a recent meta-analysis of evaluating the role of I-FABP in the diagnosis of NEC reported that the sensitivities of urinary I-FABP and the I-FABP/Cr ratio for NEC were 64% and 78% and the specificities were 73% and 75%, respectively [39]. These results showed a low sensitivity and specificity of urinary I-FBAP for NEC. Thus, there are still a lot of works that we need to do to improve its accuracy.

#### 2.2.2. Serum Amyloid A (SAA)

SAA is an acute-phase protein, rapidly synthesized by the liver and kidneys following the induction by proinflammatory cytokines. Several studies have examined levels of SAA and its association with NEC. In a recent study, the levels of urinary SAA were significantly higher in complicated NEC. An optimized cut-off value of SAA of 40.7 ng/ml was identified for the stage II and stage III NEC group by Bells' modified criteria. A cut-off value between surgical and medical NEC was determined at 34.4 ng/ml with a sensitivity of 83% and a specificity of 83%. This same group also reported that the combination of urinary SAA and serum platelet count could increase the identification sensitivity to 94% [40]. These results suggest that urinary SAA may be a potential marker in distinguishing severe NEC, particularly when applied in combination with serum platelet count.

#### 2.2.3. Prostaglandin E2 Major Urinary Metabolite (PGE-MUM)

PGE-MUM was recently reported as a possible urine biomarker of NEC given its high stability in urine. The median PGE-MUM value was highest in the NEC group (576 *μ*g/g Cre/BSA × 1000), followed by the other disease group (94 *μ*g/g Cre/BSA × 1000) and the healthy infant group (19 *μ*g/g Cre/BSA × 1000) (sensitivity: 92.3%, specificity: 81.5%; *p* < 0.01). In addition, PGE-MUM level was associated with the length of necrotic intestine and Bell's staging criteria [41]. PGE-MUM is also a biomarker of intestinal mucosal inflammation and can reflect the severity of pediatric ulcerative colitis [42]. Similar to calprotectin, PGE-MUM is not specific for NEC, thus indicating the need for additional study and exploration of possible utility when combined with other markers.

#### 2.2.4. Urinary Proteins

Liquid chromatography/mass spectrometry and enzyme-linked immunosorbent assay have been used to discover and validate various candidate biomarkers of NEC utilizing urine as a substrate. Seven urinary proteins, cluster of differentiation protein 14 (CD14), alpha-2-macroglobulin-like protein 1 (A2ML1), pigment epithelium-derived factor (PEDF), cystatin 3 (CST3), retinol-binding protein 4 (RET4), fibrinogen alpha chain (FGA), and vasolin, were utilized in various combinations to determine the presence or severity of the disease [43]. A panel consisting of A2ML1, CD14, CST3, PEDF, RET4, and VASN produced an area under the curve (AUC) of 98.4% for distinguishing medical and surgical NEC, and a panel consisting of CST3, PEDF, and RET4 produced an AUC of 98.2% for distinguishing NEC from sepsis. Together, these results suggest that NEC may be associated with several protein aberrations, and composite panels of proteins may have greater classifying capabilities than single analytes.

### 2.3. Biomarkers in Serum

#### 2.3.1. I-FABP

I-FABP exists exclusively in epithelial cells in the mucosal layer of the small intestine [44]. When intestinal epithelial cells are damaged, I-FABP proteins are released into the bloodstream. A recent longitudinal study of I-FABP compared three groups with 45 infants in each group: NEC patients, non-NEC patients, and healthy newborns. This study showed that the level of I-FABP at each time point of NEC was remarkably higher than that of the non-NEC group and the level was lowest in healthy newborns [45]. I-FABP levels are highest in the first 8 hours after symptoms occur and gradually decline with time. A cut-off value of I-FABP for NEC was determined to be 9 ng/ml in plasma and 218 ng/ml in urine 0-8 h after the onset of symptoms, and that for severe NEC was 19 ng/ml in plasma and 232 ng/ml in urine 8-16 h after the onset of symptoms [46]. Together, these results demonstrate that the level of I-FABP is dynamic, undergoing change in all groups with variability depending on the stage and time of disease diagnosis. Therefore, serum I-FABP levels and their changes may be suitable markers of early diagnosis and prognosis of NEC. There have been several meta-analyses of serum I-FABP. Yang et al. reported a sensitivity of serum I-FABP of 64% and 71% and a specificity of 91% and 76% for NEC and surgical NEC, respectively [39]. Another meta-analysis showed that the pooled sensitivity of I-FABP was 0.67 for NEC I, 0.74 for NEC II, and 0.83 for NEC III, and the pooled specificity was 0.84, respectively [47]. In summary, serum I-FABP has high specificity in the diagnosis of NEC and is a promising biomarker, but its limitation is its moderate sensitivity. Therefore, additional analytes appear to be needed to improve the sensitivity of serum I-FABP.

#### 2.3.2. Fibrinogen-*γ* Dimers

Fibrinogen-*γ* dimers and coagulant factor XIII play an important role in the prevention of blood loss and initiation of wound healing [48]. Impaired intestinal epithelial healing is a key factor in the pathogenesis of NEC. By comparing the plasma of NEC, sepsis, and healthy infants from nine institutions, Tao et al. discovered that fibrinogen-*γ* dimers were absent in patients with NEC [49]. These results provided a highly significant difference between sepsis and NEC infants (AUC = 0.958) as well as between healthy and NEC infants (AUC = 0.91). Importantly, this result was mechanistically demonstrated to be caused by a severe impairment in blood coagulant factor XIII, which covalently cross-links two molecules of fibrinogen-*γ* to produce the dimer. The levels of factor XIII were found to be lower in NEC plasma (6.56 ± 2.21 *μ*g/ml) compared with healthy infants (13.55 ± 5.38 *μ*g/ml) or the sepsis cohort (9.63 ± 4.49 *μ*g/ml). Further, the authors showed that by replacing factor XIII in NEC plasma, the FGG returned to non-NEC levels, thus providing the mechanistic understanding for the reduction in FGG dimer in NEC plasma.

#### 2.3.3. Ischemia-Modified Albumin (IMA)

Intestinal ischemia has historically been linked to the development of NEC. Under hypoxia-ischemia condition, the N-terminal amino acids of human serum albumin (HAS) are altered resulting in reduced binding capacity. Levels of this modified albumin IMA were found to be significantly higher in infants with stage III NEC than in infants with stage II NEC on the first, third, and seventh days (*p* < 0.001) [50]. IMA was also found to be superior to CRP and IL-6 in both diagnosis and follow-up of NEC progression. However, IMA has a relationship with other ischemia-related conditions, such as acute coronary syndrome and ischemia of the liver, brain, kidney, and bowel. These associations signify the lack of specificity of IMA and therefore limit the ability of IMA to distinguish NEC from these diseases.

#### 2.3.4. Interleukin-8 (IL-8)

IL-8 can be secreted by several types of cells with Toll-like receptors that are involved in the innate immune response. Benkoe et al. reported that IL-8 was significantly higher in infants with NEC compared with controls and provided a diagnostic value with an AUC of 0.99 [51]. In addition, the level of IL-8 was shown to predict the 60-day mortality in premature neonates with NEC [52]. As with most inflammatory markers that have been associated with NEC, the limitation of IL-8 is that it is a nonspecific marker for systemic inflammation, not specific to NEC.

#### 2.3.5. Inter-Alpha Inhibitor Protein (IaIp)

IaIp is considered a negative acute-phase protein that protects against the damaging effects of proteases released during acute systemic inflammation [53]. The mean IaIp levels in blood were significantly lower in infants with NEC when compared with infants with spontaneous intestinal perforation (SIP) (*p* < 0.05). ROC analysis for NEC yielded an AUC of 0.98 with a sensitivity of 100% and a specificity of 88.2% (<207 mg/l) [54]. A limitation of this study was the modest sample size. The need remains to expand the sample size to verify the accuracy and stability of IaIp for predicting NEC.

#### 2.3.6. Acylcarnitines

A recent population-based study by Sylvester et al. examined metabolic screening data of 94110 preterm neonates born in California from 2005 to 2008 [55]. All neonates included in this study had acylcarnitine measurement in birth hospitals between 12 hours and 8 days after birth. Fourteen acylcarnitine levels and ratios were found to be significantly associated with an increased risk of developing NEC upon univariate analysis. When patient characteristics and acylcarnitine levels and ratios were assessed together, five acylcarnitine levels (log C5, log C5:1, log C8:1, log C12, and log C14:1) and one acylcarnitine ratio (log FC/(C16 + C18 : 1)) were found to be significantly associated with NEC (AUC = 0.958, *p* < 0.05). Since acylcarnitine levels are derived from the metabolism of fatty and organic acids, the authors concluded that abnormal fatty acid metabolism was related to prematurity and the onset of NEC.

### 2.4. Other Noninvasive Markers to Predict NEC

#### 2.4.1. Near-Infrared Spectroscopy (NIRS)

As mentioned above, intestinal ischemia may be one of the causative factors of NEC that results in low regional tissue oxygen saturation (rSO2). NIRS measures rSO2 noninvasively. In a recent study, a continuous cerebral rSO2 ≤ 71% in the first eight hours after the occurrence of symptoms predicted the development of complicated NEC (Bell's stage 3B or death) with a sensitivity of 100% and a specificity of 80%. In this same study, the liver rSO2 ≤ 59% had a similar effect with a sensitivity of 100% and a specificity of 100%. However, NIRS monitoring did not distinguish between NEC and other intestinal diseases during the early stages of the disease [56].

#### 2.4.2. Doppler Flow Velocity

Doppler ultrasonography and flowmetry of the superior mesenteric arteries can measure the peak systolic velocity (PSV), end-diastolic velocity (EDV), resistivity index (RI), and pulsatility index (PI). Several recent publications recently demonstrated statistically significantly lower PSV (*p* = 0.001) and EDV (*p* = 0.001) in a sepsis cohort with clinical signs of NEC [57] and higher RI and PI in NEC patients compared to a control group [58]. These results suggest that intestinal blood flow ultrasonography may be a useful tool for predicting or diagnosing NEC. But like markers of ischemia discussed above, Doppler flow velocity is not specific to NEC compared with other ischemic diseases and clinical conditions that may alter intestinal blood flow patterns.

#### 2.4.3. Heart Rate Variability (HRV)

HRV can reflect the autonomic tone of neonates. It was quantified by retrieving archived electrocardiogram (EKG) data. HRV metrics showed a depression of autonomic tone that preceded the clinical NEC onset by 2 days. The pattern of HRV change was also significantly associated with the clinical severity of NEC (stage II vs. stage III) [59]. Doheny et al. conducted a prospective study using high-frequency (HF) component of heart rate variability (HRV) in the first week of life in premature infants to evaluate the utility of HF-HRV in predicting NEC. The HF-HRV power was 21.5 ± 2.7 and 3.9 ± 0.81 ms^2^ in infants that remained healthy and those that subsequently developed stage 2+ NEC, respectively (*p* < 0.001). The cut-off value of HF-HRV that predicted NEC was 4.68 ms^2^ with a sensitivity and specificity of 89% and 87%, respectively, and a positive and negative predictive value of 50% and 98%, respectively. These results suggest that low vagal tone (low HF power) in the first week of life in premature neonates may be a contributor for predicting the subsequent onset of NEC [60]. However, the HRV may be affected by many other conditions, such as feeding or pain. Therefore, the stability of HF-HRV for clinical application in NEC requires additional verification.

#### 2.4.4. Gene Polymorphisms

Gene polymorphisms are associated with many diseases including NEC. The rs1048719 polymorphism in the intron region of the GM2 activator (GM2A) gene and the rs2075783 polymorphism in the exon 1 region have been reported in association with NEC [61]. Furthermore, the rs11465996 polymorphism in the promoter region of the myeloid differential protein-2 (MD-2) gene was found to be associated with the severity of NEC. By comparing the frequency of SIGIRR variants in NEC infants to that in 20 premature infants without NEC, Sampath et al. found more SIGIRR variants in infants with NEC [62]. It remains unclear whether these associations have sufficient precision or strength of association to provide clinical utility. Moreover, since it takes a long time to obtain the results of genetic polymorphism detection, high-risk newborns should be tested shortly after birth rather than when suspected of developing NEC to compensate for time to actionable information limitation.

Although significant progress has been made in the study of NEC biomarkers, the supporting data for their clinical use remain insufficient [63], and the use of biomarkers alone to predict the risk of NEC remains of insufficient accuracy. Therefore, many scholars continue to pursue more accurate prediction models and methods. The combination of clinical parameters with biomarker analysis may significantly improve our ability to identify individuals at risk of developing NEC [64]. Since most of the existing published works on noninvasive markers provide indirect evidence of NEC, most of them do not directly reflect the degree of damage of intestinal epithelial cells. Additional progress is likely to come from a combination of markers and modalities, e.g., ultrasound and plasma or stool biomarker. Still, NEC biomarker studies to date have provided a significant increase in our understanding of its basic biology and associated pathophysiology.

## 3. Conclusions

Necrotizing enterocolitis is one of the most severe acquired diseases affecting preterm neonates. Early diagnosis remains elusive which continues to prompt human subject studies in the search for NEC-associated biomarkers that may provide for early diagnosis or the recognition of high-risk subcohorts with sufficient precision to facilitate preventive measures.

## Figures and Tables

**Table 1:** Noninvasive biomarkers of NEC.

Biomarker name	Usefulness in predicting NEC	Usefulness in differential diagnosis	Sensitivity	Specificity	Refs.	Comments
Biomarker in stool						
Calprotectin	281 *μ*g/g, early diagnosis and prediction of severity		88.24%	82.61%	20	(i) Inflammatory marker, not specific for NEC
	480 *μ*g/g		100%	84.6%	23	
	226 *μ*g/g, 299 *μ*g/g (GA < 35 w)		75%, 71%	76%, 88%	28	(ii) High individual variability
VOCs	Early prediction of NEC	NEC vs. sepsis	83%	75%	32	(iii) Larger samples will be required
Biomarker in urine						
I-FABPu	10.2 pg/nmol cr, early prediction of NEC		100%	95.6%	35	
	2.52 pg/nmol cr	NEC vs. sepsis	81%	100%	36	
			64%	73%	39	(i) Low sensitivity and specificity
	218 ng/ml (0-8 h after the onset of symptoms)	Uncomplicated vs. complicated NEC, 232 ng/ml (8-16 h after the onset of symptoms)	57%, 71%	89%, 80%	46	
Urinary SAA		34.4 ng/ml, medical vs. surgical NEC	83%	83%	40	
PGE-MUM	Severity of NEC		92.3%	81.5%	41	(ii) Not specific for NEC
Urinary proteins	CST3, PEDF, and RET4: severity of NEC	A2ML1, CD14, CST3, PEDF, RET4, and VASN: NEC vs. sepsis	89%	80%	43	(iii) High cost
			89%	90%		
Biomarker in serum						
I-FABPp	Severity of NEC		64%	91%	39	(i) Medium sensitivity
		Medical vs. surgical NEC	71%	76%		
	9 ng/ml (0-8 h after the 7onset of symptoms)	Uncomplicated vs. complicated NEC, 19 ng/ml (8-16 h after the onset of symptoms)	80%, 88%	86%, 80%	46	
	Early diagnosis		67%, 74%, 0.83%	84%	47	
Fibrinogen-*γ* dimers	Severity of NEC	NEC vs. sepsis			49	
IMA	Severity of NEC	252.57 pmol/ml, medical vs. surgical NEC	89.5%	64%	50	(ii) Not specific for NEC from other ischemic diseases
		294.91 pmol/ml, survival vs. died infants	92.9%	96.7%		
Interleukin-8	Severity of NEC	1783 pg/ml, medical vs. surgical NEC	90.5%	59.2%	52	(iii) Nonspecific marker of systemic inflammation
IaIp	207 mg/l	Spontaneous intestinal perforation	100%	88.2%	54	(iv) Larger samples will be required
Other noninvasive markers						
NIRS		Uncomplicated vs. complicated NEC			56	(i) No distinction between NEC and other intestinal diseases during the early stages
		Cerebral rSO2 ≤ 71%, liver rSO2 ≤ 59%	100%, 100%	80%, 100%		
Doppler flow velocity		NEC vs. sepsis			57	(ii) Not specific for NEC from other ischemic diseases
	RI > 0.75, PI > 1.85		96.3%, 88.8%	90.9%, 78.8%	58	
HRV	Severity of NEC (stage II vs. stage III).				59	(iii) Stability of HF-HRV requires more verification
	4.68 ms^2^ HF-HRV		89%	87%	60	
Gene polymorphisms	Severity of NEC				61	(iv) Long time to obtain the results

NEC: necrotizing enterocolitis; Ref.: reference; GA: gestational age; VOCs: volatile organic compounds; I-FABP: intestinal fatty acid-binding protein; SAA: serum amyloid A; PGE-MUM: prostaglandin E major urinary metabolite; CST3: cystatin 3; PEDF: pigment epithelium-derived factor; RET4: retinol-binding protein 4; A2ML1: alpha-2-macroglobulin-like protein 1; CD14: cluster of differentiation protein 14; VASN: vasolin; IaIp: inter-alpha inhibitor protein; IMA: ischemia-modified albumin; HRV: heart rate variability; HF: high frequency; NIRS: near-infrared spectroscopy; RI: resistivity index; PI: pulsatility index.
